# “I KNOW EVERYTHING. STOP TELLING ME I’M FINE”: A DYAD STUDY ON DISCRIMINATION AGAINST OLDER PATIENTS

**DOI:** 10.1093/geroni/igae098.0605

**Published:** 2024-12-31

**Authors:** Juncheng Tong, Jiawei Cao

**Affiliations:** Nanjing University of Chinese Medicine, Nanjing, Jiangsu, China (People’s Republic); Nanjing University of Chinese Medicine, Nanjing, Jiangsu, China (People’s Republic)

## Abstract

Despite the increasing societal attention and concern towards older patients with cancer, self-perceived social discrimination by patients, along with benevolent discrimination by their families, are often too subtle to be recognized. Since only a few studies have investigated such issues, this qualitative study aims to investigate the sources and causes of social discrimination in this group. Semi-structured interviews were conducted with 23 dyads of older patients with cancer and their primary caregivers (i.e., family members) about perceived self-discrimination and quality of life. Interview transcripts with dyads were audio recorded, transcribed verbatim, and analyzed line-by-line using thematic analysis. Two researchers independently coded transcripts and reviewed discrepancies until a consensus was reached. Four significant themes were uncovered from interviews with patients: 1) self-definition, 2) withholding information from primary caregivers, 3) socialization anxiety, and 4) stereotypes about aging and dying. Four themes emerged from interviews with primary caregivers: 1) avoiding provoking patients’ frustration and discomfort by withholding medical information, 2) being considerate but lack of communication, 3) exhaustion with repeated disappointment, and 4) family love and compromise. These findings suggest that such actions by the caregivers could result in feelings of discrimination, distrust, or even betrayal by the patients. This study provides guidance to both clinicians and caregivers to 1) identify unintended discrimination practices in healthcare, 2) strengthen dyad trust and communication, and 3) accept external support services (e.g., counseling and palliative care), which are essential for improving patients’ quality of life.

